# CD8+CXCR5+T cells infiltrating hepatocellular carcinomas are activated and predictive of a better prognosis

**DOI:** 10.18632/aging.102308

**Published:** 2019-10-30

**Authors:** Linsen Ye, Yang Li, Hui Tang, Wei Liu, Yunhao Chen, Tianxing Dai, Rongpu Liang, Mengchen Shi, Shuhong Yi, Guihua Chen, Yang Yang

**Affiliations:** 1 Department of Hepatic Surgery and Liver Transplantation Center, The Third Affiliated Hospital of Sun Yat-sen University, Guangzhou, China; 2 Guangdong Provincial Key Laboratory of Liver Disease Research, Guangzhou, China; 3 Cell-Gene Therapy Translational Medicine Research Center, The Third Affiliated Hospital of Sun Yat-sen University, Guangzhou, China

**Keywords:** hepatocellular carcinoma, tumor microenvironment, humoral immunity, cytotoxic T cells

## Abstract

CD8+ T cells are thought to be the primary cytotoxic lymphocytes exerting antitumor effects. However, few studies have focused on the antitumor effects of CD8+ T cell-mediated humoral immunity or on interactions between CD8+ T cells and B cells in hepatocellular carcinoma (HCC). We found that the frequency of IL-21-producing CD8+CXCR5+ T cells was higher in HCC tumor tissue than in peritumoral tissue or peripheral blood from the same patients or in blood from healthy donors. Moreover, CD8+CXCR5+ T cells migrated in response to supernatants from primary HCC (HCC-SN) cells, and HCC-SN cells also powerfully induced CXCR5 expression in CD8+ T cells and IL-21 expression in CD8+CXCR5+ T cells. CD8+CXCR5+ T cells from HCC patients, but not those from healthy individuals, stimulated CD19+ B cells to differentiate into IgG-producing plasmablasts. These findings reveal that CD8+CXCR5+ T cells strongly infiltrate HCC tumors, and their infiltration is predictive of a better prognosis. Surprisingly, moreover, CD8+CXCR5+ T cells produced IL-21, which induced B cells to differentiate into IgG-producing plasmablasts and to play a key role in humoral immunity in HCC.

## INTRODUCTION

Hepatocellular carcinoma (HCC) accounts for approximately 70%-80% of all primary liver cancer cases and is characterized by high mortality and poor survival rates [1, 2]. This is in part because therapeutic options currently remain limited. Recent studies have found that immune system dysregulation plays a crucial role in the development of HCC [3, 4]. Tumor-infiltrating immune cells, including cytotoxic T cells, CD4+ helper T cells, myeloid-derived suppressor cells (MDSCs), and regulatory B cells, all exert effects that could potentially influence the progression of HCC. Consequently, immunotherapy is a promising method for the treatment of HCC [5]. And because there is a correlation between the density of tumor-infiltrating lymphocytes within HCC lesions and prognosis, the recommendation for immunotherapy is based on the presence of a high density of tumor-infiltrating T cells [6, 7]. Therefore, a better understanding of the phenotype, regulation, and function of tumor-infiltrating cytotoxic T cells in HCC is required for development of these therapies.

CD8+CXCR5+ T cells have been found in T cell lineage acute lymphocytic leukemia [8], pancreatic tumors [9] and colorectal tumors, as well as nearby lymph nodes [10]. These T cells exhibit high functionality, and their presence is predictive of a better prognosis. Compared to CD8+CXCR5- T cells, CD8+CXCR5+ T cells produce different levels of interferon (IFN)-γ and tumor necrosis factor (TNF)-α during infection with lymphocytic choriomeningitis virus (LCMV) [8] or HIV [11], and in chronic infections [12]. Moreover, CD8+CXCR5+ T cells account for approximately 25% of all CD3+ T cells, which support B cell activation, affinity maturation, and isotype switching within the follicles of secondary lymphoid organs [12, 13]. These characteristics of CD8+CXCR5+ T cells demonstrate their potential capacity for antitumor activity; thus, the phenotype, regulation and function of CD8+CXCR5+ T cells in HCC should be evaluated, especially in the context of humoral immune responses. In the present study, therefore, we examined CD8+CXCR5+ T cells found within matched tumor tissue, peritumoral tissue, and peripheral blood from HCC patients. Our findings reveal that intratumoral CD8+CXCR5+ T cells are an indispensable part of humoral antitumor immunity.

## RESULTS

### High infiltration of CD8+CXCR5+ T cells into HCC tumors predicts a better prognosis

**Figure 1:**
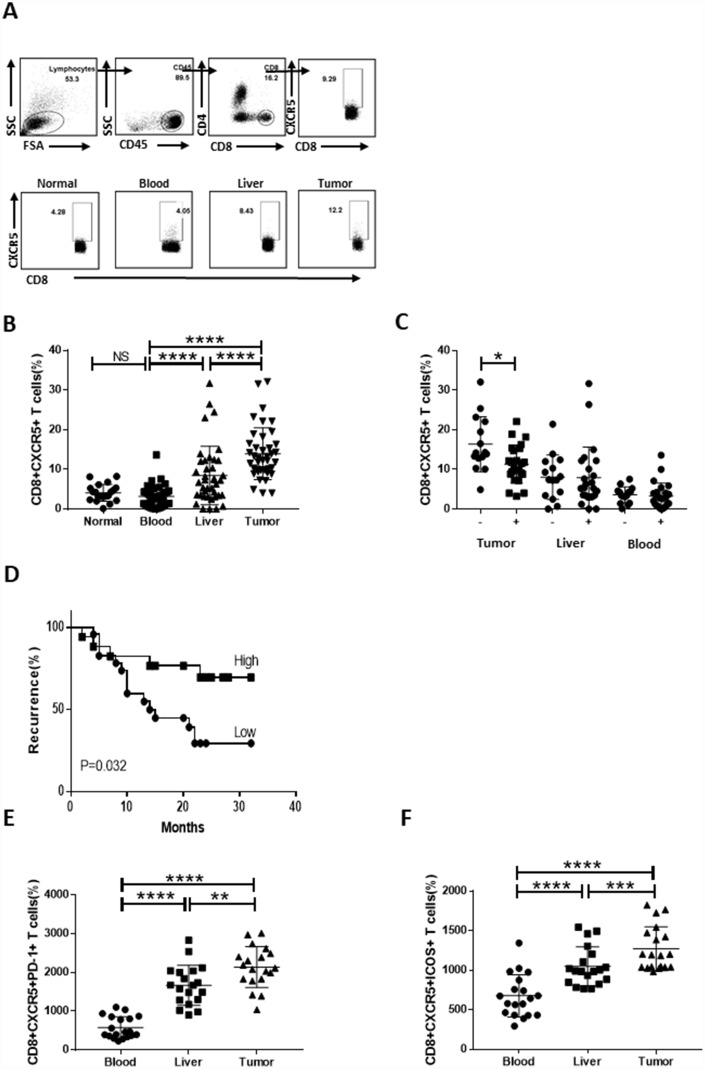
**Strong infiltration of CD8+CXCR5+ T cells into HCC tumors predicts a better prognosis.** Fresh samples were stained with anti-CD8, anti-CXCR5, anti-PD-1 and anti-ICOS antibodies. (**A**–**B**) Following gating of CD8+ T cells, the frequencies of CD8+CXCR5+ T cells from healthy PBMCs (n=20), and matched HCC tumor tissue, peritumoral liver tissue and PBMC samples (n=40) were analyzed. (**A**) One representative experiment is shown. (**C**) Association of tumor-infiltrating CD8+CXCR5+ T cells with microvascular invasion (n=25 for positive, n=15 for negative) is shown. (**B**–**C**) The data indicate the median with the interquartile range. (**D**) Patients were divided into two groups (Low/High) based on the median of the tumor-infiltrating CD8+CXCR5+ T cell percentages. The early recurrence rate was compared between the two groups using the log-rank test. (**E**–**F**) PD-1 and ICOS expression by CD8+CXCR5+ T cells differed among tumor tissue and matched peritumoral tissues and peripheral blood (n=19). The data indicate the median with the interquartile range. *P<0.05, **P<0.01, ***P<0.001 and ****P<0.0001 determined using the Mann-Whitney U test (**B**, **C**, **E** and **F**).

To assess the presence of CD8+CXCR5+ T cells within HCC tumors, we used flow cytometry to analyze the CD8+CXCR5+ T cell content in 20 healthy blood samples and 40 HCC specimens (Table 1), each of which included blood, peritumoral liver, and tumor tissue samples the same patient. Regardless of whether blood or tumor was assessed, the majority of CD8+ T cells were CXCR5-, though a clear CD8+CXCR5+ T cell population was detected. In HCC patients, the percentage of CD8+CXCR5+ T cells was obviously larger in tumor tissue than in the peritumoral liver or blood. There was no significant difference in the numbers CXCR5+ cells among circulating CD8+ T cells in HCC patients and healthy donors (Figure 1A and 1B). Importantly, the percentage of tumor-infiltrating CD8+CXCR5+ T cells correlated negatively with microvascular invasion (Figure 1C) and early recurrence (Figure 1D and Table 2). Moreover, the levels of PD-1 and ICOS markers expressed by CD8+CXCR5+ T cells were significantly higher in tumor tissue than in the matched blood or peritumoral liver tissues or in healthy blood samples (Figure 1E and 1F and Supplementary Figure 1). Together, these data demonstrate that CD8+CXCR5+ T cells strongly infiltrated HCC tumors and predicted a better prognosis.

**Table 1:** Characteristics of the study population (N=40).

**Variable**	**HCC (N=40)**
Age (years old)	50.80±11.164
Gender (Male/ Female)	35/5
HBV-DNA (<1*e^2^ vs ≥1*e^2^)	18/22
TNM Stage (I/II/III/IV)	14/6/15/5
Tumor Differentiation (I/II/III/IV)	6/20/9/5
Tumor Multiplicity (multiple/ solitary)	15/25
Tumor Size, cm	4.95±2.363
Tumor Microvascular Invasion (No/Yes)	18/22
AFP (<400/≥400)	18/22

AFP: alpha-fetoprotein; TNM: tumor, node, metastases.

**Table 2:** Univariate and multivariate analysis of the prognostic factors for recurrence-free survival and overall survival (N=40).

**Variable**	**Recurrence-free survival**			
	**Univariate**		**Multivariate**	
	**HR (95%CI)**	**P value**	**HR (95%CI)**	**P value**
Age (years old)	1.020(0.976-1.067)	0.382		
Gender (Male vs Female)	0.667(0.154-2.891)	0.589		
Tumor Multiplicity (multiple vs solitary)	0.454(0.132-1.565)	0.211		
Tumor Size, cm (>5 vs ≤5)	0.936(0.769-1.139)	0.509		
Tumor Differentiation (III+IV vs I+II)	0.826(0.400-1.703)	0.604		
Tumor Microvascular Invasion (yes vs no)	2.311(0.906-5.896)	**0.080**	2.376(0.929-6.080)	0.071
TNM Stage (III+IV vs I+II)	0.621(0.248-1.556)	0.309		
AFP (<400 vs ≥400)	1.087(0.412-2.867)	0.867		
HBV-DNA (<1*e^2^ vs ≥1*e^2^)	1.301(0.522-3.239)	0.572		
CD8+CXCR5+	3.144(1.101-8.975)	**0.032**	3.222(1.125-9.231)	**0.029**

AFP: alpha-fetoprotein; TNM: tumor, node, metastases.

### Strong infiltration of IL-21-producing CD8+CXCR5+ T cells in HCC correlates with disease stage

Numbers of IL-21-producing CD8^+^CXCR5^+^ T cells are significantly increased in chronic hepatitis B (CHB) [14].

**Figure 2:**
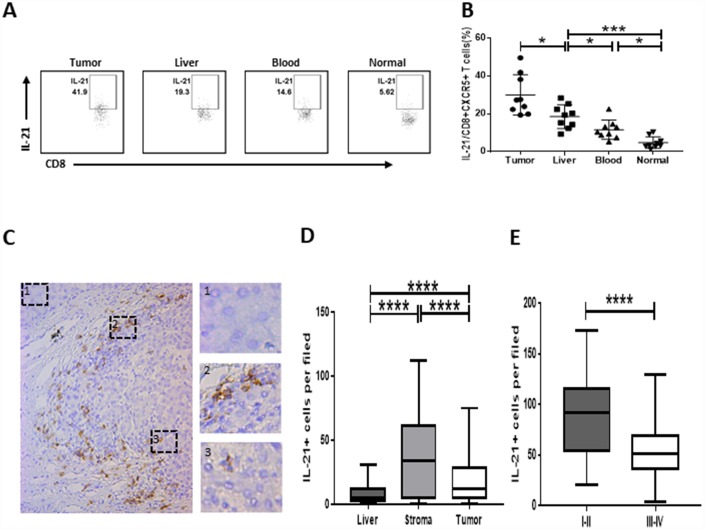
**Strong infiltration of IL-21-producing CD8+CXCR5+ T cells in HCC correlates with disease stage.** (**A**–**B**) Flow cytometric analysis of IL-21 production by CD8+CXCR5+ T cells (n=9). The cells were characterized using FACS with sequential gating of lymphocyte cells, CD45+ cells and then CD8+CXCR5+ cells. (**A**) One representative experiment is shown. (**B**) The data indicate the median with the interquartile range. (**C**–**E**) Immunohistochemical staining of IL-21+ cells in paraffin-embedded HCC tissue (n=96). The distribution of IL-21+ cells is shown in (**C** and **D**). Micrographs at higher magnification show the stained peritumoral liver (1), peritumoral stromal region (2), and cancer nest (3). The association of the density of tumor-infiltrating IL-21+ cells with the TNM staging of patients is shown in (**E**). *P<0.05, **P<0.01, ***P<0.001 and ****P<0.0001 determined using Mann-Whitney U test (**B** and **D**) or student’s t test (**E**).

We investigated whether IL-21 was produced by CD8+CXCR5+ T cells in HCC. We found that numbers of IL-21-producing CD8+CXCR5+ T cells were significantly higher within HCC tumor tissue than in the matched blood and peritumoral liver tissue or in healthy blood samples (Figure 2A and 2B). We subsequently found that strong tumor infiltration by IL-21-producing CD8^+^CXCR5^+^ T cells led to the accumulation of IL-21+ cells within the peritumoral stroma and that this accumulation correlated negatively with the TNM staging of the patients (Figure 2C–2E).

### Migration and induction of CD8+CXCR5+ T cells by HCC-SN

**Figure 3:**
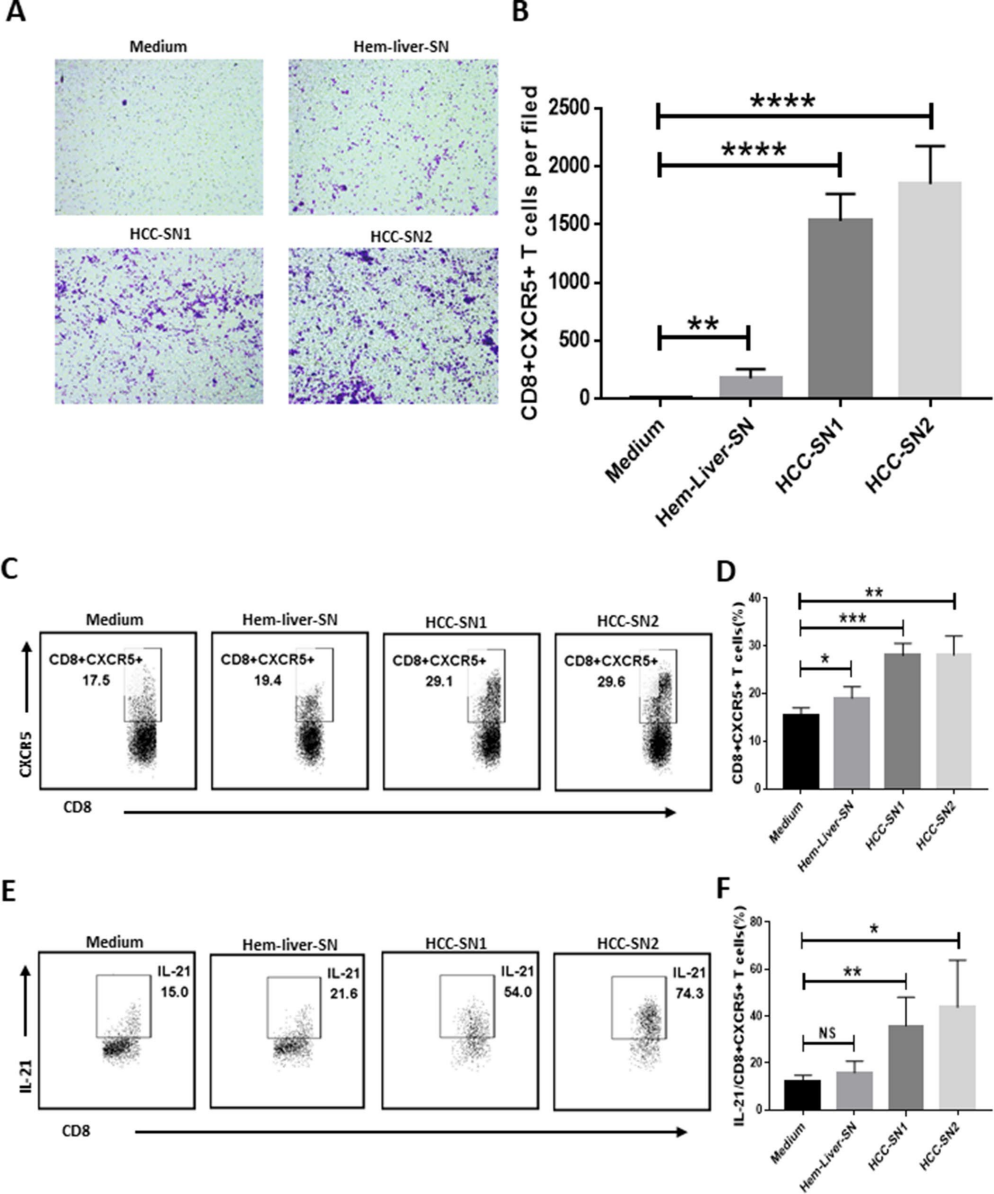
**Differentiation and chemotaxis of CD8+CXCR5+ T cells is induced by HCC-SN cells.** (**A**–**B**) Culture supernatants from primary HCC cell cultures (HCC-SN1 and HCC-SN2 cells), but not normal liver cultures (Hem-liver-SN cells), induce chemotaxis of healthy blood CD8+CXCR5+ T cells sorted by FACS (n=3). (**A**) One representative experiment is shown. (**B**) The data indicate the mean±SD. (**C**–**E**) HCC-SN1 and HCC-SN2 cells, but not Hem-liver-SN cells, are able to induce the CD8+CXCR5+ or IL-21+ CD8+CXCR5+ phenotype in healthy blood CD8+ T cells (n=3). The results shown represent four separate experiments. The data indicate the mean±SD. *P<0.05, **P<0.01, ***P<0.001 and ****P<0.0001 determined using student’s t test (**B**, **D** and **F**).

Having established the presence of an antitumor CD8+CXCR5+ T cell subset in HCC, we next performed experiments to investigate whether environmental factors facilitated the migration and induction of CD8+CXCR5+ T cells. Culture supernatant from primary HCC tumor (HCC-SN) cells, but not from normal liver cells, showed a great ability to induce chemotaxis in CD8+CXCR5+ cells from healthy blood (Figure 3A and 3B). Moreover, the addition of HCC-SN cells to cultures of CD8+ T cells from healthy blood induced a considerable proportion of CD8+CXCR5+ T cells (Figure 3C), and upregulated IL-21 production by CD8+CXCR5+ T cells (Figure 3D). By contrast, culture with supernatant from normal liver cells failed to augment CXCR5 or IL-21 expression in CD8+ T cells.

### Tumor-infiltrating CD8+CXCR5+ T cells from HCC patients were potent inducers of plasmablasts in vitro

**Figure 4:**
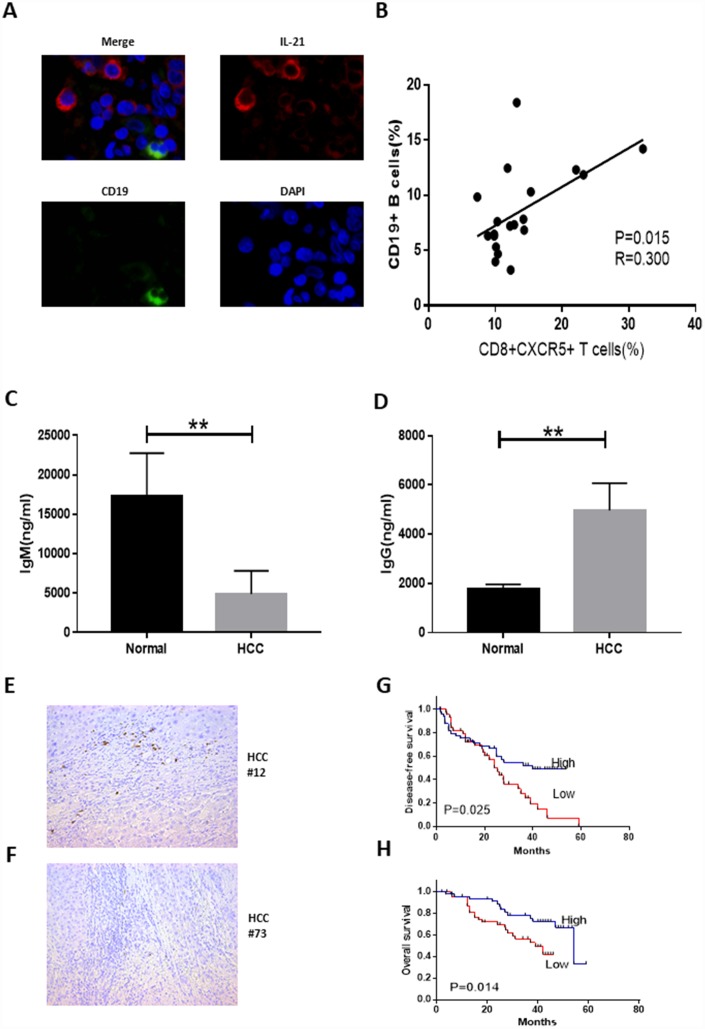
**Tumor-infiltrating CD8+CXCR5+ T cells from HCC patients are potent inducers of plasmablasts differentiation in vitro.** (**A**) Representative immunofluorescence images of CD19 (green), IL-21 (red) and nuclear staining with DAPI (blue) in HCC tissue. (**B**) Associations between tumor-infiltrating CD8+CXCR5+ T cells and tumor-infiltrating CD19^+^ B cells (n=19). (**C**–**D**) Coculture of autologous CD19+ B cells with healthy blood CD8+CXCR5+ T cells or tumor-infiltrating CD8+CXCR5+ T cells. On day 5, the supernatants were harvested. IgG (**C**) and IgM levels (**D**) were determined using an ELISA (n=3). (**E**–**F**) Immunohistochemical staining of CD138+ B cells in paraffin-embedded HCC tissue (n=96). (**G**–**H**) Patients were divided into two groups (Low/High) based on the median of the tumor-infiltrating CD138+ B cell percentages. The DFS and OS curves between the two patient groups were compared using the log-rank test. **P<0.01.

We demonstrated that large numbers of IL-21-producing CD8+CXCR5+ T cells accumulate within HCC tumor tissue. Because IL-21 plays a critical role in regulating immunoglobulin production [15, 16], we investigated the possible effects of tumor-infiltrating CD8+CXCR5+ T cells on B cells. Confocal microscopy showed that IL-21+ T cells and CD19+ B cells were located in the same region (Figure 4A). In HCC patients, moreover, there was a correlation between the frequencies of CD19+ B cells and CD8+CXCR5+ T cells (Figure 4B). These data demonstrate that immunoglobulin produced by B cells recruited by CD8+CXCR5+ T cells may play an important role in antitumor activity.

In addition, coculture of CD8+CXCR5+ T cells with CD19+ B cells led to B cell class switching, and IgM and IgG levels in the supernatant differed between HCC patients and healthy donors (Figure 4C and 4D). These results indicate that CD8+CXCR5+ T cells in HCC patients can provide helper B cells and induce immunoglobulin-producing plasmablasts. We subsequently examined the relationship between CD138+ expression and HCC patient survival (Figure 4E–4H). HCC patients who underwent curative resection, and for whom follow-up data were available, were divided into two groups based on the median CD138+ plasma cell density (n=96, Table 3). This revealed obvious positive associations between CD138+ plasma cell density and both disease-free survival (DFS) (n=96, p=0.025, Figure 4G) and overall survival (OS) (n=96, p=0.014, Figure 4H). Univariate and multivariate analyses revealed that tumoral CD138 expression was an independent prognostic factor for DFS and OS (Table 4).

**Table 3:** Characteristics of the study population (N=96).

**Variable**	**HCC (N=96)**
Age (years old)	49.70±10.944
Gender (Male/ Female)	87/9
HBV-DNA (<1*e^2^ vs ≥1*e^2^)	19/77
TNM Stage (I/II/III/IV)	54/11/25/6
Tumor Differentiation (I/II/III/IV)	18/36/27/15
Tumor Multiplicity (multiple/ solitary)	8/88
Tumor Size, cm	4.71±2.794
Tumor Microvascular Invasion (No/Yes)	67/29
AFP (<400/≥400)	17/79

AFP: alpha-fetoprotein, TNM: tumor, node, metastases.

**Table 4:** Univariate and multivariate analysis of the prognostic factors for recurrence-free survival and overall survival (N=96).

**Variable**	**Disease-free survival**				**Overall survival**			
	**Univariate**		**Multivariate**		**Univariate**		**Multivariate**	
	**HR (95%CI)**	**P value**	**HR (95%CI)**	**P value**	**HR (95%CI)**	**P value**	**HR (95%CI)**	**P value**
Age (years old)	0.764 (0.436-1.340)	0.348			0.992 (0.493-1.996)	0.982		
Gender (Male vs Female)	1.213 (0.482-3.053)	0.681			0.902 (0.450-1.808)	0.771		
Tumor Multiplicity (multiple vs solitary)	1.138 (0.536-2.416)	0.736			1.252 (0.481-3.257)	0.646		
Tumor Size, cm (>5 vs ≤5)	1.353 (0.780-2.347)	0.282			1.876 (0.935-3.761)	**0.076**	2.207 (1.087-4.479)	**0.028**
Tumor Differentiation (III+IV vs I+II)	0.648 (0.325-1.294)	0.219			0.895 (0.368-2.176)	0.807		
Tumor Microvascular Invasion (yes vs no)	1.245 (0.699-2.218)	0.457			1.664 (0.800-3.464)	0.173		
TNM Stage (III+IV vs I+II)	1.086 (0.587-2.011)	0.793			1.481 (0.698-3.142)	0.306		
AFP (<400 vs ≥400)	0.513 (0.269-0.977)	**0.042**	0.558 (0.320-0.972)	0.063	1.030 (0.489-2.172)	0.938		
HBV-DNA (<1*e^2^ vs ≥1*e^2^)	0.943 (0.547-1.625)	0.832			1.078 (0.543-2.141)	0.830		
CD138+	0.531 (0.304-0.925)	**0.025**	0.541 (0.283-1.033)	**0.039**	0.393 (0.186-0.830)	**0.014**	0.345 (0.161-0.741)	**0.006**

AFP: alpha-fetoprotein; TNM: tumor, node, metastases.

## DISCUSSION

Tumor-infiltrating immune cells and the status of the tumor microenvironment profoundly influence prognosis in human malignancies [17, 18]. Most studies to date have focused on CD8+ CTL cells as important effectors in the antitumor cellular immune response because of their ability to release cytotoxic molecules to induce cell death. Hence, most current immunotherapeutic approaches involve enhancement of CD8+ T cell activity through blockade of immune checkpoint inhibitors [19]. We focused on the CD8+CXCR5+ T cell population in HCC patients because this cell subtype, especially in the tumor microenvironment, has not been investigated in HCC and was found to possess high functionality in colorectal cancer [10] and LCMV infection [8]. Our results show that CD8+CXCR5+ T cells strongly infiltrate the tumor tissue and play a key role in the HCC-directed antitumor immune response. Tumor-infiltrating CD8+CXCR5+ T cells also highly express PD-1 and ICOS, which may indicate that blocking CD8+ T cell immune checkpoint inhibitors could serve as a therapeutic option in the future.

In addition to cellular antitumor immunity, humoral immunity also impacts tumor progression [20, 21]. Few studies have investigated whether CD8+ T cells plays a role in humoral immunity, especially in HCC. We found that IL-21-producing CD8+CXCR5+ T cells strongly infiltrate HCC tumor tissue as compared to peritumoral tissue or peripheral blood from the same patients or blood from healthy donors. Consistent with an earlier study [22], we found that IL-21+ cells infiltrate the peritumoral and tumor tissues and may participate in antitumor immunity.

The tumor microenvironment is regarded as a major site of tumor antigen presentation and adaptive antitumor immunity maturation, which includes cross-talk between immune cells. CXCL13, the corresponding ligand for CXCR5, has been detected in tumor cells, dendritic cells, and T follicular helper cells as well as stromal cells in B cell follicles [23]. Primary HCC-SN, but not hem-liver-SN, showed a great ability to induce CD8+CXCR5+ cell chemotaxis. Moreover, primary HCC-SN may provide a variety of tumor antigens, molecules, proteins, and microRNAs to mediate induction of CD8+CXCR5+ T cells and IL-21 production.

Several recent reports indicate that IL-21 plays a critical role in T cell-dependent and T cell-independent human B cell terminal differentiation [15, 16, 24]. Importantly, although IL-21 is mainly secreted by T follicular helper cells [25], we found that tumor-infiltrating CD8+CXCR5+ T cells are able to induce B cells to differentiate into IgG-producing plasmablasts. In human tumors, the process of B cell maturation is complicated and sophisticated [22]. In addition to IL-21, TLR9-CpG ODN interaction [26], BCR engagement, and co-stimulation via CD40L [24] play essential roles in human B cell differentiation during generation of humoral immune responses. This suggests that CD8+CXCR5+ T cells interact with B cells in HCC patients, and their role in the complicated process of B cell differentiation warrants further investigated.

In summary, we observed first that strong infiltration of CD8+CXCR5+ T cells into HCC tumor tissue reduces the likelihood of recurrence. Second, strong accumulation of IL-21-producing CD8+CXCR5+ T cells stimulates B cells to differentiate into IgG+ plasmablasts in the HCC microenvironment. Third, the presence of CD138+ plasmablasts was predictive of longer DFS and OS in 96 HCC patients. In the future, therapies aimed at boosting interaction between antitumor CD8+CXCR5+ T cells and B cells may be developed to provide a novel approach to HCC treatment.

## METHODS

### Patients and specimens

Tissue-infiltrating leukocytes were isolated from fresh tumor tissue, peritumoral liver tissue, and peripheral blood samples obtained from patients who underwent curative resection at the Third Affiliated Hospital of Sun Yat-sen University between September 2016 and June 2018 (Table 1). Tissues for immunohistochemistry (IHC) or immunofluorescence (IF) were also obtained from patients receiving surgical resection at the same hospital between September 2011 and December 2012 (Table 3). All of these patients had HBV infection. None had previously received any anticancer therapy. Patients with HIV infection or other autoimmune disease or cancer were excluded. The clinical stages of the tumors were classified according to the guidelines of the International Union Against Cancer. Written informed consent was obtained from all patients, and the protocols were approved by the Review Board of the Third Affiliated Hospital of Sun Yat-sen University.

### Isolation of immune cells from peripheral blood and tissue

Peripheral blood mononuclear cells (PBMCs) were isolated using a standard Ficoll procedure. Tissue-infiltrating leukocytes were dissociated from tissue specimens and collected as described previously [27]. Briefly, specimens were cut into small pieces and digested in RPMI 1640 (Gibco) supplemented with 100 μg/ml Liberase TL (Roche), 100 μg/ml DNase I (Sigma-Aldrich) and 20% FBS (Gibco) for 30 min at 37°C. The homogenates were filtered through a 150-mm cell strainer and separated by Ficoll density gradient centrifugation (Axis-Shield). The isolated PBMCs were washed with Hanks’ balanced salt solution and resuspended in RPMI 1640 supplemented with 10% FBS.

### Flow cytometric analysis of cell surface molecule expression

Cells were stained with anti-CD4-APC-CY7, anti-CD8-FITC, anti-CXCR5-APC, anti-PD-1-PE, and anti-ICOS-PECF594 (all from Biolegend), after which they were washed and marker expression was detected using flow cytometry (BD LSR2).

### Flow cytometric analysis of intracellular cytokine expression

PBMCs from healthy individuals and HCC patients were cultured in a 96-well plate (1×10^6^ cells/well) in 500 μl of culture medium. The cells were stimulated with PMA (50 ng/ml, Sigma-Aldrich) and ionomycin (400 ng/ml, Sigma-Aldrich) for 1 h at 37°C. Brefeldin A (10 lg/ml, Sigma-Aldrich) was then added, and the cells were incubated for another 4 h. Thereafter, the samples were fixed, permeabilized and stained with anti-CD4-APC-CY7, anti-CD8-FITC, anti-CXCR5-APC, anti-PD-1-PE, anti-ICOS-PECF594 (all from Biolegend), and anti-IL-21-BV421 (BD Biosciences). Cytokine-producing cells were detected using flow cytometry (BD LSR2).

### Preparation of culture supernatant conditioned by primary HCC tumor cells

Tumor specimens of HCC patients or healthy liver specimens from hemangioma patients (normal liver tissue far away from the hemangioma) without concurrent autoimmune disease or HBV, HCV, HIV, or syphilis infection were completely digested and then washed with medium containing polymyxin B (20 g/mL; Sigma-Aldrich) to exclude endotoxin contamination. A total of 1×10^7^ dissociated cells were resuspended in 10 mL of complete medium and cultured in 100-mm dishes. After 2 days, the supernatants were harvested, centrifuged, and stored at -80°C.

### Cell migration assay

Migration of CD8+CXCR5+ T cells was assayed using a 24-well Transwell plate with a 6.5-μm pore polycarbonate membrane insert (Corning, USA) after the cells were purified using a FACS ARIA cell sorter (BD). The inserts were loaded with CD8+CXCR5+ T cells (5×10^5^ cells in 100 μl serum-free medium) and placed into the 24-well plates containing 600 μl of HCC-SN, Hem-liver-SN or serum-free medium. After incubating for 3 h at 37 °C, the migrated cells were fixed with 100% methanol for 30 min and then stained with 0.1% crystal violet (Leagene, China) for 15 min. To evaluate chemotaxis, the number of migrated cells were counted under a light microscope (Leica, Germany).

### Separation of human CD19+ B or CD8+ T cells from peripheral blood

PBMCs were isolated from peripheral blood samples from healthy donors by Ficoll centrifugation (Axis-Shield). CD19+ B or CD8+ T cells (collected populations were more than 95% CD19+ or CD8+) were sorted from among the PBMCs using a MACS CD19+ B or CD8+ T cell Isolation Kit (Miltenyi Biotech).

### TFH-B cell culture

PBMCs and TILs were labeled with anti-CD8-FITC and anti-CXCR5-APC (both from Biolegend), after which CD8+CXCR5+ T cells were purified using a FACS ARIA cell sorter (BD). The purity was higher than 98%. A total of 2×10^4^ T cells were cocultured with the same number of CD19+ B cells in a total volume of 200 μl of Gibco 1640. The supernatant was harvested on day 5 and stored at -20 °C. IgG and IgM levels in the supernatant were measured using an ELISA according to the manufacturer’s instructions (R&D).

### Statistical analysis

Results are expressed as the mean±SD or median with interquartile range. Statistical comparisons were made as indicated in the figure legends using two-sided tests. Group data were analyzed using Student’s *t* test or log-rank test for normally distributed variables, and the Mann-Whitney U test was used for non-parametric comparisons. Correlations between two parameters were assessed using Pearson correlation analysis. Multivariate analysis of the prognostic factors for OS and DFS was performed using the Cox proportional hazards model and log-rank test. Cumulative survival time was assessed using the Kaplan-Meier method. Values of P<0.05 were considered significant.

### Ethics approval

The biopsy specimens were obtained under protocols approved by the ethics committees of The Third Affiliated Hospital of Sun Yat-sen University and informed consent was obtained from all patients.

## Supplementary Material

Supplementary Figure 1

Supplementary Table 1
