# Supplementary material for: CD8+CXCR5+T cells infiltrating hepatocellular carcinomas are activated and predictive of a better prognosis
Source: Aging (Albany NY). 2019 Oct 30;11(20):8879–91. doi: 10.18632/aging.102308 (PMC6834425; doi:10.18632/aging.102308)
Supplement: Supplementary Figure 1 [file aging-11-102308-s001.pdf]

SUPPLEMENTARY FIGURE

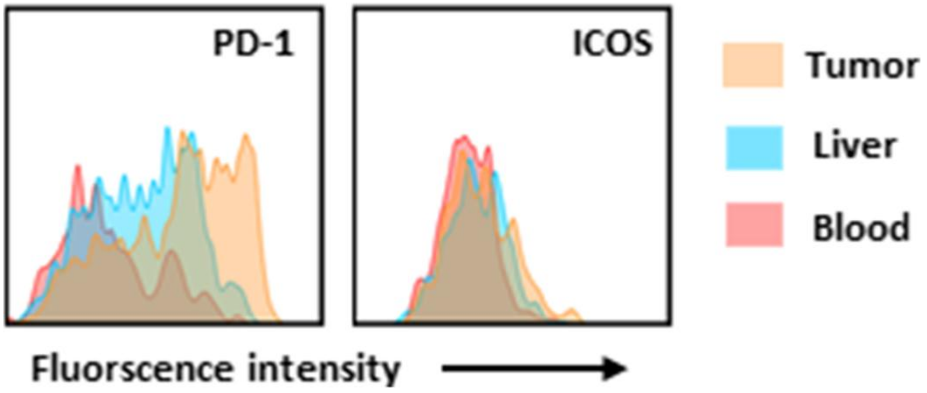

Supplementary Figure 1. PD-1 and ICOS expression by CD8+CXCR5+ T cells differed among the tumor tissue and matched peritumoral tissues and peripheral blood from the same patients.
