# Supplementary material for: CD8+CXCR5+T cells infiltrating hepatocellular carcinomas are activated and predictive of a better prognosis
Source: Aging (Albany NY). 2019 Oct 30;11(20):8879–91. doi: 10.18632/aging.102308 (PMC6834425; doi:10.18632/aging.102308)
Supplement: Supplementary Table 1 [file aging-11-102308-s002.pdf]

SUPPLEMENTARY TABLE

Supplementary Table 1. Characteristics of the both study population.

Variable	HCC (N=96)	HCC (N=40)	P value
Age (years old)	49.70±10.944	50.80±11.164	0.477
Gender (Male/ Female)	87/9	35/5	＜0.005
HBV-DNA (<1*e2 vs ≥1*e2)	19/77	18/22	0.626
TNM Stage (I+II/III+IV)	65/31	21/19	0.072
Tumor Differentiation (I+II/III+IV)	54/42	26/14	0.345
Tumor Multiplicity (multiple/ solitary)	8/88	15/25	＜0.005
Tumor Size, cm	4.71±2.794	4.95±2.364	0.614
Tumor Microvascular Invasion (No/Yes)	67/29	18/22	＜0.005
AFP (<400/≥400)	17/79	18/22	＜0.005

AFP: alpha-fetoprotein; TNM: tumor, node, metastases.
